# Engineering Performance Evaluation of Recycled Red Mud Stabilized Loessial Silt as a Sustainable Subgrade Material

**DOI:** 10.3390/ma15093391

**Published:** 2022-05-09

**Authors:** Qianwei Ma, Wei Duan, Xiaofeng Liu, Peiying Fang, Ruifeng Chen, Tingyuan Wang, Zirui Hao

**Affiliations:** 1 College of Civil Engineering, Taiyuan University of Technology, Taiyuan 030024, China; maqw2019@163.com (Q.M.); liuxiaofeng02@tyut.edu.cn (X.L.); fangpeiying0380@link.tyut.edu.cn (P.F.); wty1011@126.com (T.W.); haozirui0398@link.tyut.edu.cn (Z.H.); 2 Institute of Geotechnical Engineering, Southeast University, Nanjing 211189, China; 15735651673@163.com

**Keywords:** red mud, loessial silt, mechanical properties, resistivity, hydraulic conductivity

## Abstract

Industrial solid waste red mud discharge has caused serious environmental problems. This study utilized red mud as an additive to loessial silt being used for roadway subgrade material. In this study, unconfined compressive test, direct shear test, electrical resistivity test, and hydraulic conductivity test were conducted on red mud stabilized loessial silt (RMLS) with different red mud dosage (*D*_R_) to investigate *D*_R_ effect on mechanical-electrical-hydro properties. Scanning electron microscope (SEM) and X-ray diffraction (XRD) analyses were carried out to reveal the mechanism from micro perspective. The results showed addition of appropriate amount of red mud (30–42%) effectively improved unconfined compressive strength of treated loessial silt but reduced resistivity and hydraulic conductivity. Significant correlation between resistivity and strength performance of RMLS mixture was developed. Microscopic analysis indicates red mud addition will promote generation of hydration products such as calcium silicate hydrations (C-S-H), calcium silicate aluminates hydrations (C-A-S-H), and ettringite (Aft), which will tightly connect surrounding particles of loessial silt and hydrates. Red mud particles adhere to surface of soil particles and fill in pores between them improving a compact and stable structure. This study demonstrated the feasibility of using red mud as a stabilization material for roadway subgrade and proved that resistivity measurement is a nondestructive testing method to evaluate mechanical properties for RMLS mixture.

## 1. Introduction

Loessial silt is widely distributed in China, in which the proportion of silt is the highest (accounts for more than 50% of total particle quality) and thus its properties are mainly determined by silt [1,2]. However, the water cementation of silt is weak owing to its characteristics of low plasticity index, loose, permeable, and low cohesion [3,4], which will have a negative impact on engineering projects. Since silt is liquefiable [5,6] and silt filled subgrade presents difficult compaction but easy hydration [7], there are hazards of subgrade deformation when using silt alone in subgrade. Loessial silt is a particularly special silt with collapsibility leading to a sharp decrease of strength and a sharp increase in deformation as water content increases [8,9], which further increase the likelihood of collapse when using loessial silt in subgrade. Therefore, it is necessary to improve loessial silt to meet the engineering requirements. Solidification treatment is one main method to improve soft soil subgrade and foundation [10]. It is common to use lime curing agent in engineering [11,12] and formed lime soil is widely used in subgrade engineering projects. However, there are some weaknesses of lime soil which are low frost resistance, low early strength, high drying and temperature shrinkage, and easy cracking [13,14]. Therefore, researchers attempted to improve the performance of lime soil by adding some additives, such as metakaolin [15], slag [16], and their mixtures [17].

**Figure 1:**
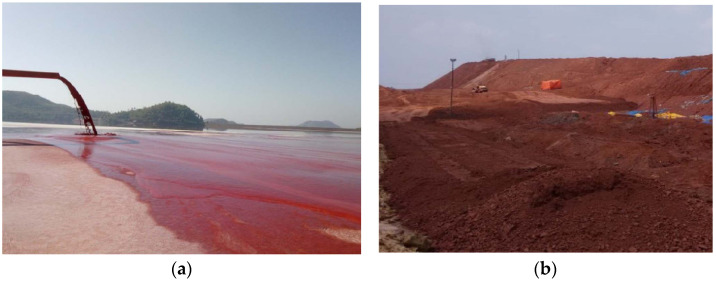
Discharge and stacking of red mud. (**a**) Mud treatment. (**b**) Dry mud treatment.

Red mud is a very fine particle and strong alkaline solid waste generated by the extraction of alumina from bauxite and the production of 1 t alumina generates 1–2 t red mud [18]. Discharge and storage of red mud occupies land, increases likelihood of soil and groundwater pollution, and requires high maintenance costs (see Figure 1). Therefore, it is urgent to accelerate the comprehensive utilization of red mud. At present, red mud waste has been used alone [19] or combined with lime-fly ash [20], desulfurized gypsum [21], cement kiln dust [22], and ground granulated blast-furnace slag (GGBS) [23] for replacing natural soil in subgrade to improve weak soil strength.

However, using red mud as a raw material for subgrade is expensive and it replaces all-time available soil [24]. Thus, utilization of red mud as an additive for subgrade soil is another solution to refining subgrade properties and at the same time reducing environment pollutions [21,24]. Actually, several studies have begun to explore the feasibility of red mud as an additive material for road construction. Sridevi et al. [25] improved expansive soil with lime-stabilized red mud and conducted compaction and unconfined compression strength (UCS) tests on the soil samples. UCS value increased from 16.8 to 100.6 kN/m^2^ with 30% lime-stabilized red mud content upon curing for 90 days. Sabat and Mohanta [26] conducted UCS and soaked California bearing ratio (CBR) tests on stabilized expansive soil to study stabilizing effect of a red mud-dolime fine-fly ash cushion. It was reported that the optimum percentages of dolime fine and fly ash for forming cushion were 15% and 8% respectively. Hu et al. [27] synthesized red mud and fly ash to form a geopolymer as a stabilizing agent for pavement base materials. The results showed UCS of geopolymer stabilized samples increased from 10.5 MPa to 16.1 MPa with the increase in temperature from 20 to 38 °C at standard ambient temperature. Li et al. [28] demonstrated the significant improvement of fluidity and compressive strength in red mud-slag-based geopolymeric grouting material with the influence of superplasticizer, which facilitated the large-scale utilization of red mud in cementitious materials.

Past studies rarely investigated the application of red mud waste as an additive to subgrade soil and the published work only focused on mechanical performance of the stabilized soil (for example, [25]). Loessial silt is commonly used for road subgrade in northwest China but there are limited records of adding red mud to loessial silt. Therefore, this study will analyze comprehensive properties of stabilized loessial silt and provide a theoretical reference for the application of red mud waste to improve loessial silt roadbed, which exhibits both environmental and economic benefits.

The objective of this study is to investigate the effect of red mud dosage (*D*_R_) on the mechanical-electrical-hydro properties of loessial silt and reveal effect mechanism of red mud from micro perspective. First, the optimal moisture content (*w*_opt_) and maximum dry density (*ρ*_dmax_) of red mud stabilized loessial silt (RMLS) for sample preparation were obtained through compaction test. Second, the improvement of strength performance of the sample was evaluated through unconfined compressive test, direct shear test, and electrical resistivity test. Then hydraulic conductivity test was performed to study the change of permeability of the sample with different *D*_R_ values. Curing age was also found to be correlated with UCS and hydraulic conductivity coefficient. The strength prediction method based on the measurement of resistivity was developed meanwhile. Finally, the effect mechanism of red mud was revealed by microscopic analysis. 

## 2. Materials and Methods

### 2.1. Materials

#### 2.1.1. Loessial Silt and Lime

**Figure 2:**
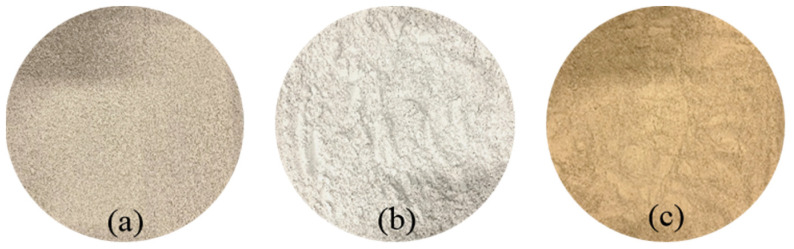
Test materials, (**a**) red mud, (**b**) lime, (**c**) loessial silt.

The soil sample selected in this study was typical loessial silt in Taiyuan, Shanxi province (see Figure 2). The basic physical indexes of silt are shown in Table 1. The proportion of CaO content in lime used in this study is 98%, which meets the requirement of test specification for inorganic binder stabilized materials of Highway Engineering [29].

#### 2.1.2. Red Mud

**Figure 3:**
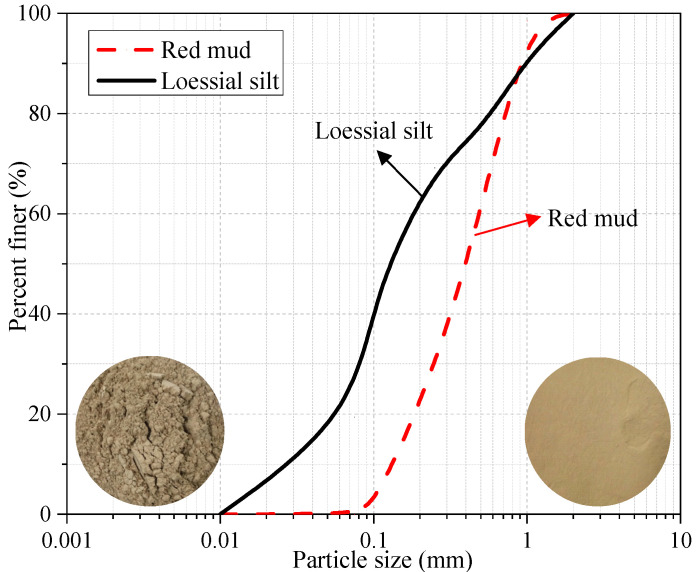
Particle size distribution of red mud and loessial silt.

The red mud waste (see Figure 2) generated through Bayer process in this study was from an aluminum plant in Liulin, Shanxi province. Basic physical properties and main chemical compositions of red mud are shown in Table 2 and Table 3 respectively. Figure 3 illustrated the particle size distribution curve. The unconfined compressive strength (UCS) value of red mud is about 0.7–0.8 MPa but it will collapse completely after immersion owing to poor water stability. Table 4 showed leaching toxicity concentration results of red mud and the concentration of harmful elements in red mud leaching solution meets the prescribed standard [30].

**Figure 4:**
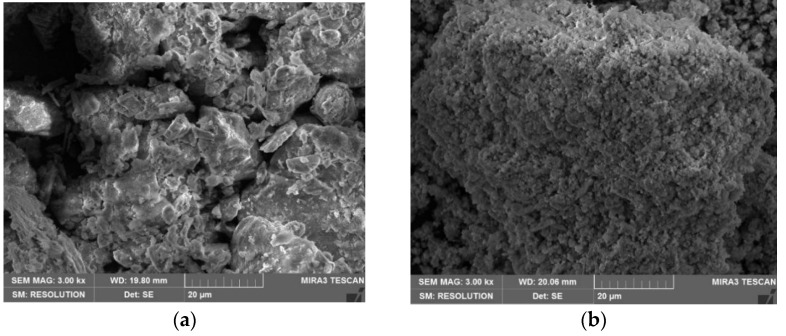
The SEM images (3.00 k×) of (**a**) loess, (**b**) red mud.

Figure 4 shows the scanning electron microscope (SEM) images of the red mud and loess. An aggregate structure composed of massive fine particles in red mud can be observed from Figure 4b, which does not exist in loess (Figure 4a). Red mud has a larger specific surface area and a higher density [31].

#### 2.1.3. Sample Preparation

Loessial silt and red mud waste were crushed, dried, selected by 1-mm sieve, dried in an oven, and sealed for storage. The ratio of lime to loessial silt was fixed at 1:10 in which mass fraction of lime is 10% of the dry mass of loessial silt. *D*_R_ proportion for RMLS mixture accounted for 0%, 6%, 12%, 24%, 30%, 36%, 42%, 48%, and 54% respectively. Mix loessial silt, lime and red mud using each ratio as shown in the Table 5 and then stir the mixture. Note that before sample preparation, the optimal moisture content (*w*_opt_) and maximum dry density (*ρ*_dmax_) of RMLS samples were obtained based on compaction tests (see Table 5) and samples of each ratio were prepared with the *w*_opt_ value. 

Then the RMLS samples were prepared in a 50 mm × 50 mm hydraulic jack (diameter × height) using static compaction method. After stripping the samples, they were placed in a safety plastic bag for sealing and numbered. Subsequently, the samples were maintained in a curing room with standard constant temperature ((20 ± 2) °C) and humidity (95%) for different curing ages (3, 7, 14, 21, and 28 days). On the last day, samples were immersed into a water tank in which the water surface level was around 2.5 cm higher than the top of samples for 24 h until each designed curing age is complete [32], which is then used for UCS, resistivity, and hydraulic conductivity test. Finally, some of the samples were obtained to be immersed in anhydrous ethanol and then dried in the air for SEM and XRD analysis.

### 2.2. Test Methods

#### 2.2.1. Compaction Test

The standard light compaction test was adopted to evaluate the compatibility and obtain the *w*_opt_ value and the *ρ*_dmax_ value of RMLS samples of each mixing ratio. The sample mixed at desired moisture was divided into three parts and placed in a metal cylinder respectively for compaction; compaction is 592.2 kJ/m^3^ per unit volume of the hammer. Then moisture content and density of the sample were measured and a compaction curve was obtained through repeating the same procedure at five moisture content levels.

#### 2.2.2. Unconfined Compressive Test

The unconfined compressive test was conducted by microcomputer controlled-electronic universal testing machine (WDW-100) (produced by Chenda Technology company, Jinan, China). The sample was placed in the center of the lower bearing platform of the universal testing machine and the position of the upper pressing plate was adjusted to contact with the upper surface of the sample. The descending speed of the upper plate was controlled by displacement with loading rate of 1 mm/min and pressurization process stops until the sample has a through crack completely losing its bearing capacity. The unconfined compressive strength (UCS) was obtained. Moreover, electrical resistivity test could also be performed simultaneously to explore correlation between the strength and resistivity of the sample.

#### 2.2.3. Electrical Resistivity Test

**Figure 5:**
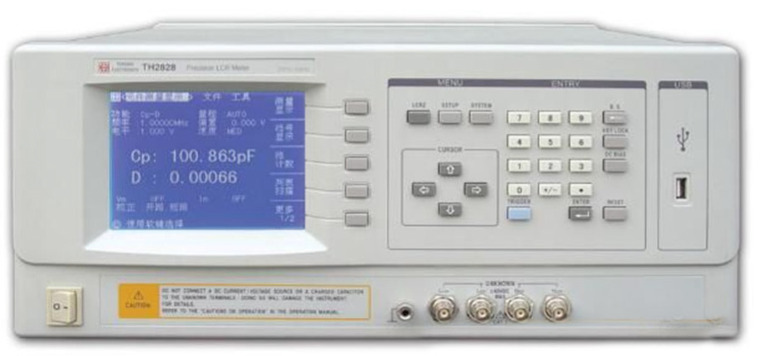
Model TH2828A of LCR digital bridge.

Model TH2828A of LCR digital bridge (produced by Tonghui Electronics company, Changzhou, China) (Figure 5) was used in this study. The sample whose two sides were covered with a graphite layer evenly was placed between two copper electrode pieces. After the electrode pieces were connected with the digital bridge, resistance (*R*) of the sample was tested. The resistivity (*ρ*) could be calculated using the Formula (1).

(1) $$\rho =R\times S/L\times 10^{-3}$$

where *ρ* is resistivity (Ω·m), *R* is resistance (Ω), S is contacted surface area between electrode and the sample (mm^2^), and L is distance between two electrodes pieces (mm). Resistivity at the current frequency of 50 kHz was used for analysis in the following sections.

#### 2.2.4. Direct Shear Test

**Figure 6:**
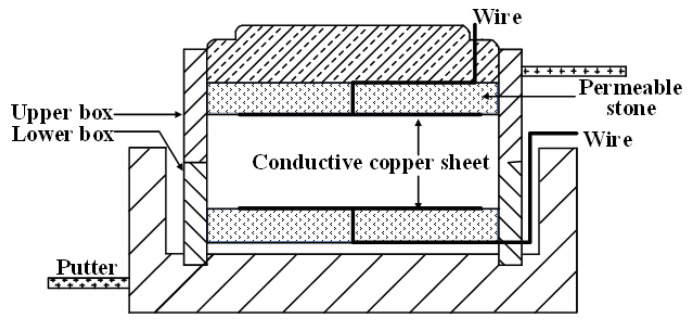
Schematic diagram of new direct shear box.

To study the resistivity change during direct shear test process, a new direct shear box was developed. The copper sheets were pasted on upper and lower permeable stones and contacted with sample surface. Wires passed holes of permeable stones and were welded with copper sheets. The other side of wire was connected with LCR digital bridge to record resistivity change of sample during the shear process (see Figure 6). The direct shear tests were carried out under vertical pressure of 100, 200, 300, and 400 kPa respectively.

#### 2.2.5. Hydraulic Conductivity Test

The device used in the hydraulic conductivity test was PN3230 flexible wall permeameter (produced by GEOEQUIP, Orlando, FL, USA). After installing the sample, water injection and back pressure saturation were carried out to remove pore gas from the sample. Then osmotic pressure was set to 50 kPa, 100 kPa, 150 kPa, 200 kPa, and 250 kPa respectively, and permeation rate of the sample was recorded [33]. 

#### 2.2.6. Microscopic Test

**Figure 7:**
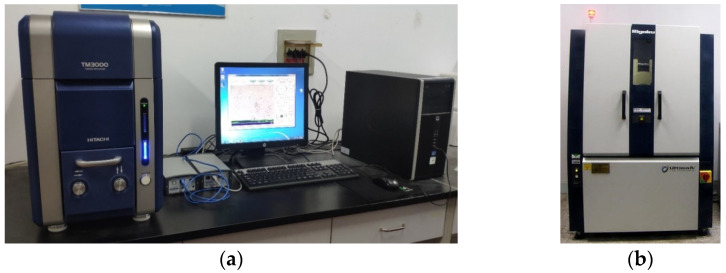
Microanalysis test. (**a**) Scanning electron microscope. (**b**) X-ray diffraction.

Scanning electron microscope (SEM) of TM 3000 (produced by Hitachi Limited, Tokyo, Japan) and X-ray diffraction (XRD) tests of Ultima Ⅳ X-ray diffractometer (produced by Rigaku, Tokyo, Japan) (Figure 7) were carried out to study micro effect mechanism of RMLS. In SEM test, the air-dry samples were scanned with a 2*θ* ranging from 10° to 90°. The micro morphology of soil particles and hydrate could be clearly observed at 500 times using Axio-Scope-Al (produced by Zeiss, Jena, Germany). Formation of new phase could be judged through XRD test.

## 3. Results

### 3.1. The Effect of D_R_ on UCS

**Figure 8:**
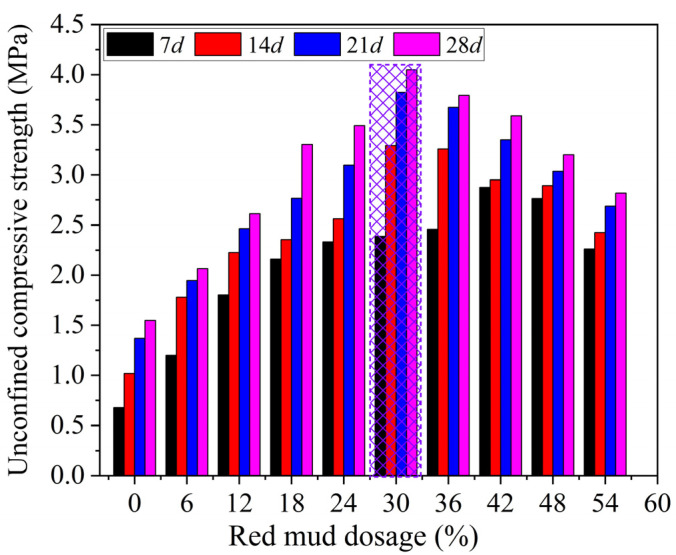
UCS of loessial silt stabilized with different red mud dosage at different curing ages.

Figure 8 shows the effect of *D*_R_ on UCS value (*q*_u_) of RMLS at different curing ages. It can be seen that the *q*_u_ first increases and then decreases with the increase of *D*_R_ at each curing age, which means that red mud addition can enhance the UCS of the RMLS mixture when *D*_R_ is less than the optimal red mud dosage (*D*_R0_). For example, *q*_u_ of loessial silt at the curing age of 28*d* is 1.55 MPa, while it increases to 4.05 MPa (1.6 times of 1.55 MPa) at the same curing age after adding red mud of *D*_R_ = 30% (marked by grid in Figure 8). A theory can explain such a result that the hydrolysis reaction and strong alkalinity of red mud can promote the formation of cementitious hydrates which will enhance the UCS of RMLS. Note that *q*_u_ will decrease slightly when the *D*_R_ exceeds *D*_R0_, which is because excess ions dissolved from red mud will hinder hydrolysis reaction of red mud and superfluous red mud will damage the integrity of the soil structure leading to a decrease of UCS for RMLS mixture. The *D*_R0_ values of RMLS mixture at the curing ages of 7*d*, 14*d*, 21*d*, and 28*d* are 42%, 30%, 30%, and 30% respectively (marked with the grid). All tested RMLS samples meet the subbase working condition requirements of class II/III Highway (1.5–2.0 MPa) and class I highway/Expressway (1.5–2.5 MPa) (RISN-TG003-2007, China) except RMLS with *D*_R_ = 6% at the curing age of 7*d*.

**Figure 9:**
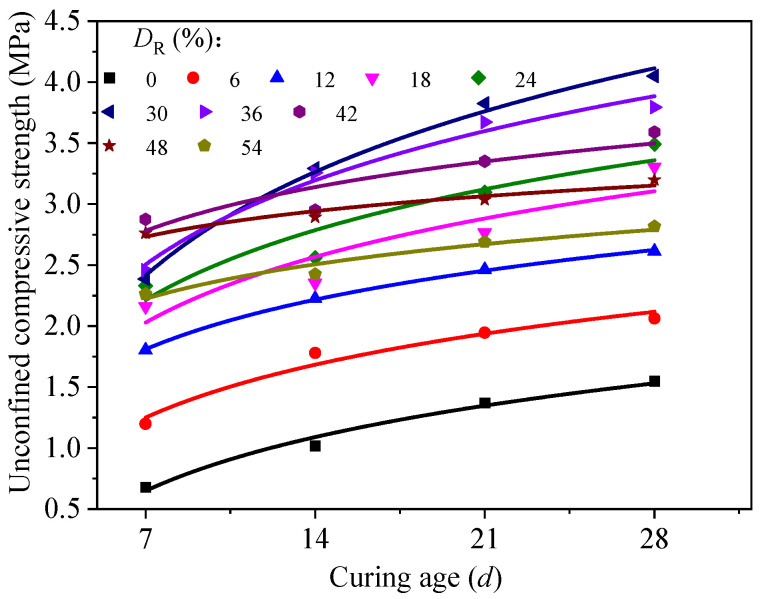
Relationship between unconfined compressive strength and curing age.

Moreover, UCS of RMLS increases as the curing age increases with each fixed *D*_R_. Figure 9 shows a positive correlation between *q*_u_ and curing period of RMLS that there is an increasing trend of *q*_u_ with the increase of curing age when *D*_R_ is fixed. To be more specific, the UCS of RMLS mixture exhibits a reasonably good logarithmical correlation with curing age, which can be expressed as:

(2) $$q_{u}=a\ln\left(t\right)+b$$

where *a* and *b* are fitting parameters that represent the change rate of UCS and the UCS value at the curing age of 1*d*, respectively. Values of *a* and *b* of each red mud dosage are summarized in Table 6 based on the test results.

In Figure 9, UCS of RMLS mixture increases rapidly in the early curing age but tends to be stable later, which can be explained by the hydration rate change of active components in RMLS mixture during the curing process. In the early curing age, Ca^2+^ from lime (Ca(OH)_2_) continuously hydrolyzes and reacts with SIO_4_^4−^, AlO^2−^, and AlSiO^4−^ dissolved in red mud to generate new cementitious substances. These cementitious substances turn into the skeleton of RMLS mixture through filling in the internal pores of RMLS mixture immediately and enhancing the compactness of RMLS mixture. In this case, UCS of RMLS mixture is improved rapidly. However, as the curing age increases, internal pores are filled with cementitious substances and hydration reaction rate is gradually weakened, which slows down the increasing rate of UCS of RMLS mixture until it stops.

**Figure 10:**
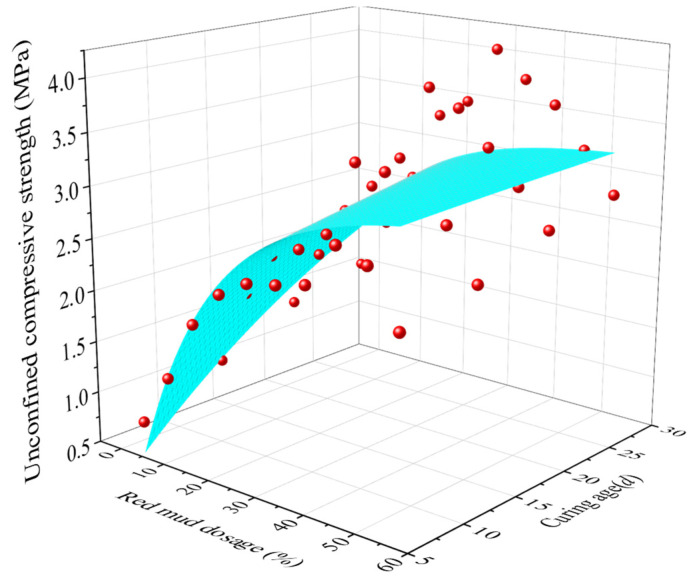
Fitting figure of UCS with different *D*_R_ and curing age.

Furthermore, a fitted equation based on an exponential 2D function is built for UCS value (*q*_u_) of RMLS mixture with *D*_R_ and curing age (*t*) (see Figure 10), which can be expressed by:

(3) $$q_{u}=q_{u0}+B\times exp\left\{-D_{R}/C\right\}\times exp\left\{-t/D\right\}$$

in which *q*_u0_, *B*, *C*, and *D* represent the fitted coefficient and parameter values are shown in Table 7. This equation with high correlation coefficient will provide a critical guidance for selection of engineering parameters and prediction to UCS when applying RMLS mixture to subgrade filling in loess regions.

### 3.2. The Effect of D_R_ on Shear Property

**Figure 11:**
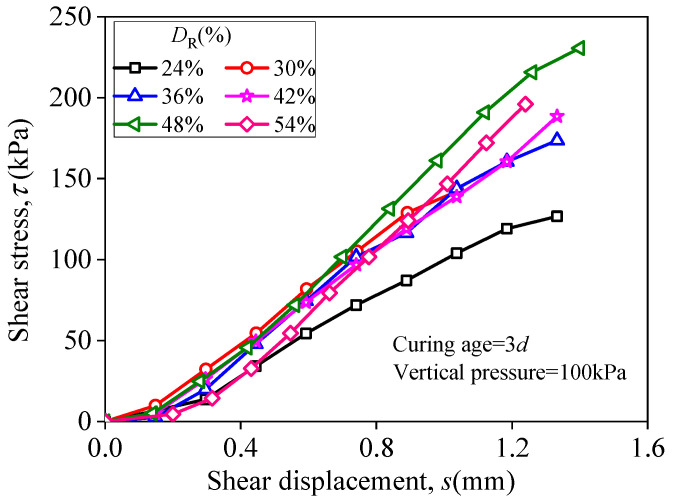
Relationship between shear stress and shear displacement.

Figure 11 displays curves of shear stress of RMLS mixture and shear displacement with each different *D*_R_ (curing age is 3*d* and vertical pressure is 100 kPa). It is apparent that as shear displacement increases, shear stress increases slowly first, then there is a linear pattern increase in a specific interval, and finally the increasing rate slows down gradually until the sample is broken. Actually, shear stress has no obvious change first, since particle structure is loose. After particle structure is squeezed tightly, there is a linear increase of shear stress owing to the increased contacted area of the sample particles. Then with further increase of shear displacement, shear plane of the sample decreases, which means that the shear stress needed to overcome the connection of soil particles decreases and thus increasing trend of shear stress tends to be stable until the sample is broken. 

Furthermore, the effect of *D*_R_ on shear stress of RMLS is consistent with UCS, that is the shear stress first improved and weakened later at each fixed shear displacement with the increase of *D*_R_. However, note that the effect of *D*_R_ on shear stress is less compared to the effect on UCS when shear displacement is small (when *s* < 0.6 mm).

**Figure 12:**
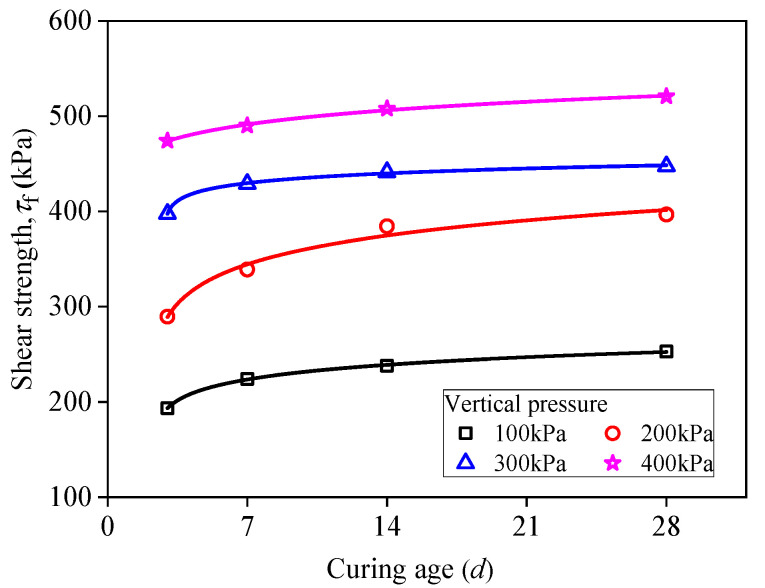
Relationship between shear stress and curing age (*D*_R_ = 30%).

Figure 12 presents the relationship between shear strength and curing age under different vertical pressures (*D*_R_ = 30%). It can be observed that the shear strength of RMLS mixture is increased with the increase of applied vertical pressure when curing age is fixed. Specifically, RMLS structure is loose and easy to be sheared in the early curing age. With the increase of curing age, hydrolysis reactions of RMLS mixture are promoted to generate cementitious products, which will fill in the pores of RMLS and make the structure denser. Therefore, shear strength of RMLS mixture is increased accordingly. This process is similar with the increase process of UCS described before.

### 3.3. The Effect of D_R_ on Resistivity

**Figure 13:**
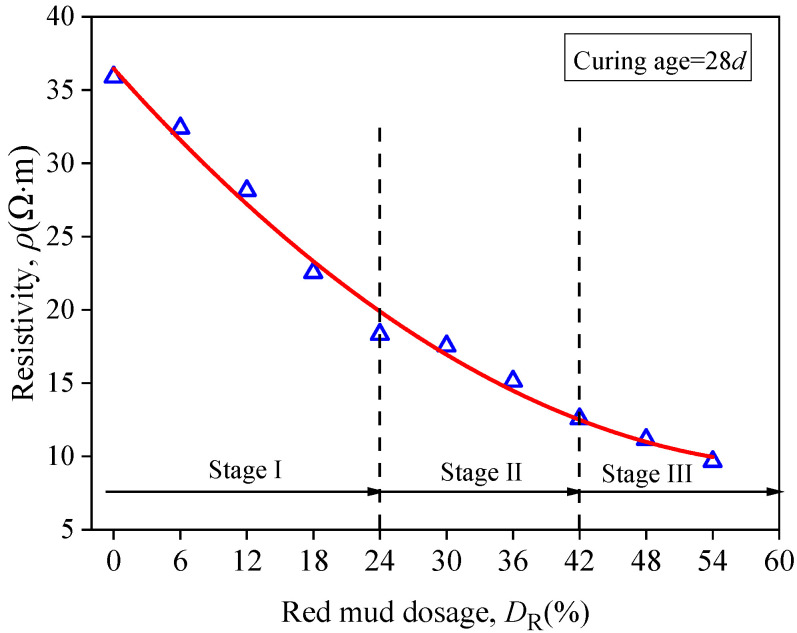
Relationship between resistivity and red mud dosage.

Figure 13 illustrates the relationship between resistivity of RMLS mixture and *D*_R_ at the curing age of 28*d*. It can be observed that resistivity of RMLS mixture showed the maximum value when *D*_R_ = 0 (loessial silt) and decreases with the increase of *D*_R_. The descending process of resistivity can be divided into three stages: stage I (0% < *D*_R_ < 24%), in which the resistivity decreases rapidly and *D*_R_ has a significant effect on the resistivity change; (2) stage II (24% < *D*_R_ < 42%), the decreasing trend slows down and effect *D*_R_ is reduced; (3) stage III (42% < *D*_R_ < 54%), the resistivity tends to be a constant value and *D*_R_ has little influence on it.

The *w*_opt_ value of RMLS mixture increases as the *D*_R_ increases, and pore moisture content increases accordingly since volume of sample is fixed. In stage I, charged particles and clay particles dissolve in red mud because of its good hydrophilic capacity, which enhances the conductivity of RMLS mixture and reduces the resistivity significantly. As *D*_R_ increases, stage II begins, in which the concentration of dissolved ions reduces and thus the decreasing rate of resistivity is less than stage I. In stage III, the concentration of dissolved ions remains at a constant value such that the decreasing trend of resistivity is gradually weakened and tends to be stable finally.

**Figure 14:**
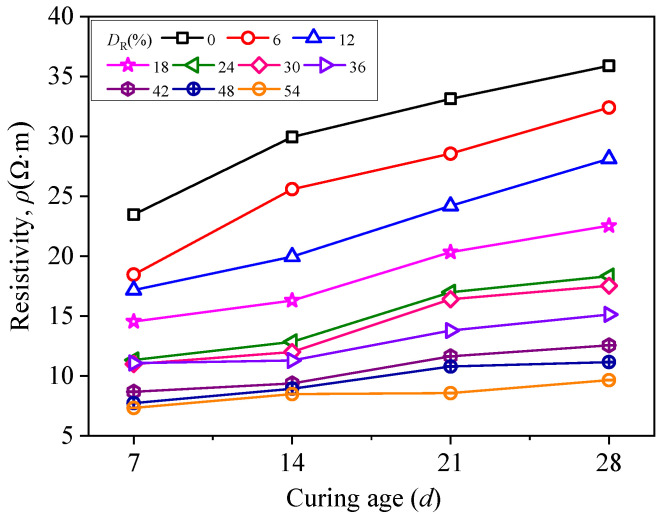
Relationship between resistivity and curing age.

Figure 14 shows the relationship between resistivity of RMLS mixture and curing age. It is evident that the resistivity of RMLS mixture with each *D*_R_ increases with the increase of curing age. When 0% < *D*_R_ < 18%, the resistivity increases steadily as the curing age increases. However, when 24% < *D*_R_ < 54%, there is no significant change in resistivity in the early curing age (*t* < 14*d*); however, a rapid increase is seen later (*t* > 14*d*).

**Figure 15:**
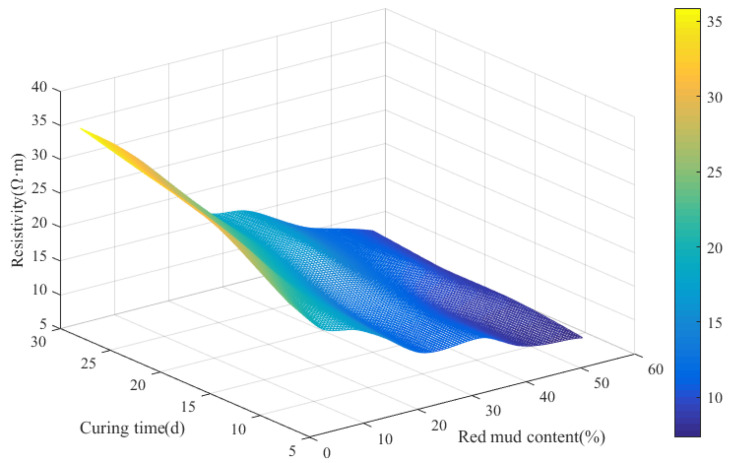
Three-dimension surface diagram of the correlations between red mud dosage, curing time, and electrical resistivity.

The three-dimension response surface diagram for resistivity of RMLS mixture with *D*_R_ and curing age is displayed in Figure 15 to explore the relationship between resistivity of RMLS mixture, red mud dosage, and curing age. Apparently, the decreasing trend of amplitude of resistivity is significant when *D*_R_ is less than 42%. However, with a further increase in *D*_R_ (*D*_R_ > 42%), morphology of the surface diagram becomes flat indicating the negligible influence of *D*_R_ on resistivity. Increasing trend for amplitude of resistivity as the increase of curing age is generally steady but the increased amplitude is less than the decreased amplitude of *D*_R_, which means effect of curing age on resistivity is less significant than *D*_R_.

### 3.4. The Effect of D_R_ on Hydraulic Conductivity

**Figure 16:**
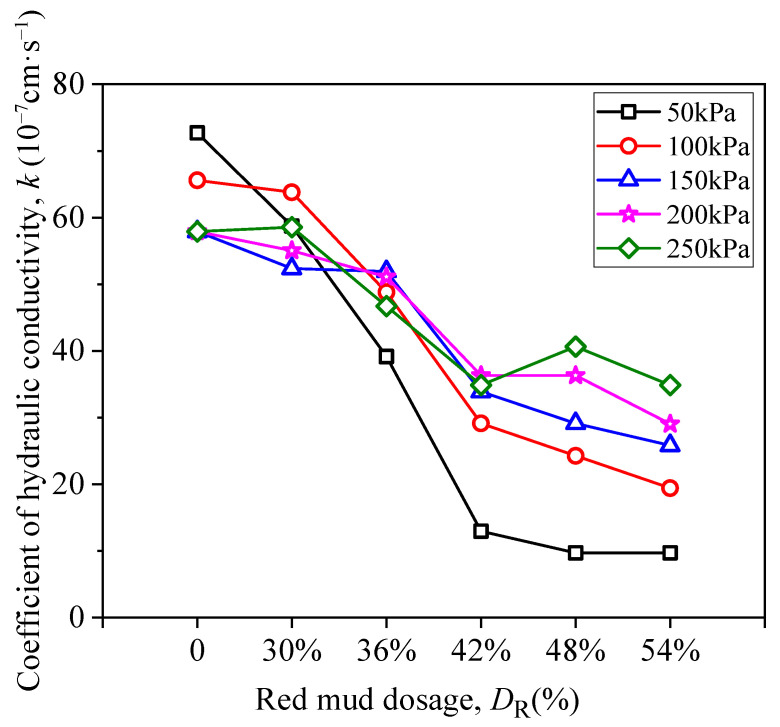
Relationship between coefficient of hydraulic conductivity and red mud dosage.

Figure 16 presents the correlation between coefficient of hydraulic conductivity (*k*) and *D*_R_ under different pressure (curing age is 7*d*). It can be observed that the *k* value of loessial silt (*D*_R_ = 0%) is 7.3 × 10^−6^ cm/s but decreases to 9.7 × 10^−7^ cm/s (nearly 10 times decrease) when *D*_R_ = 54% under osmotic pressure of 50 kPa. Apparently, red mud can improve anti-permeability property of loessial silt effectively. The phenomenon of hydration reaction of red mud, lime, and silt can explain this result, such that with the addition of red mud, more cementitious material generated from hydration reaction will block seepage channel and therefore improve anti-permeability property of RMLS mixture.

**Figure 17:**
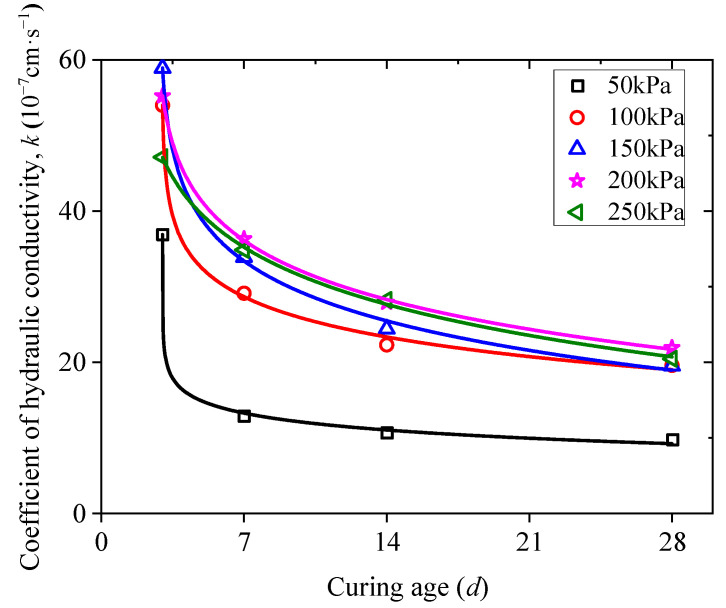
Relationship between hydraulic conductivity coefficient and curing age at different osmotic pressures (*D*_R_ = 42%).

Furthermore, Figure 17 shows the correlation between *k* of RMLS mixture and curing age when *D*_R_ is 42%. It is evident that *k* value decreases rapidly in the early curing age but tends to be constant later, which is because the hydration reaction rate is first high but later decreases with the increase of curing age (similar with the description of *D*_R_ effect on hydraulic conductivity). An expression based on logarithmic function was built to represent the relationship between *k* and curing age for RMLS mixture:

(4) k=A+B×ln(t+C)

where *A*, *B*, *C* are fitting parameters as shown in the Table 8 based on test result related to osmotic pressure and curing age and t is the curing age.

### 3.5. Correlation between Resistivity and Strength Parameter

#### 3.5.1. UCS

**Figure 18:**
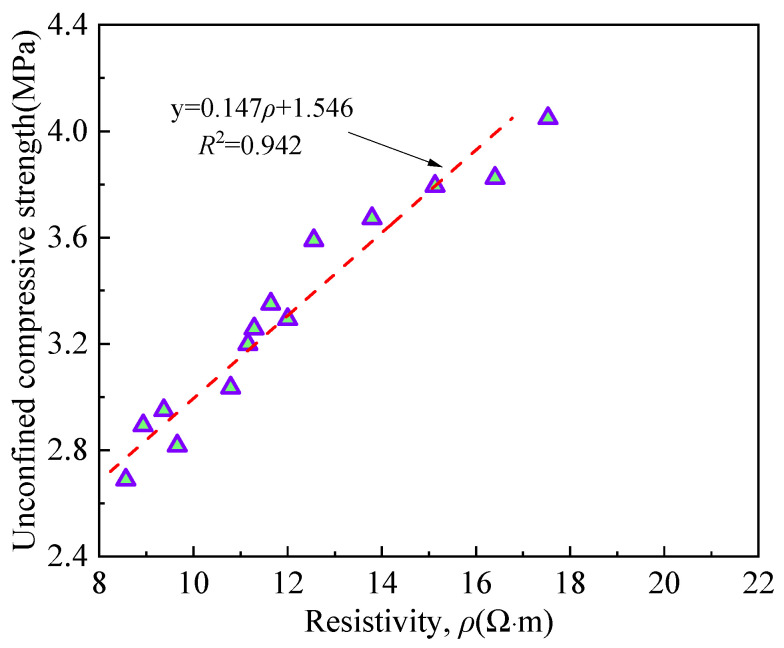
Relationship between resistivity and unconfined compressive strength.

A significant correlation between UCS and resistivity of RMLS mixture could be predicted based on above analysis. Note that UCS values are obtained under the condition of *D*_R_ = *D*_R0_, which varies with the change of curing age. Figure 18 shows the relationship between UCS and resistivity for RMLS mixtures, in which a good linear correlation is derived. Hydration gels and red mud fine particles have filling effects on soil, which will increase the resistivity. Meanwhile these filling effects can also improve soil structure to a stable and compact structure and thus increasing the UCS of soil. 

#### 3.5.2. Direct Shear Parameter

**Figure 19:**
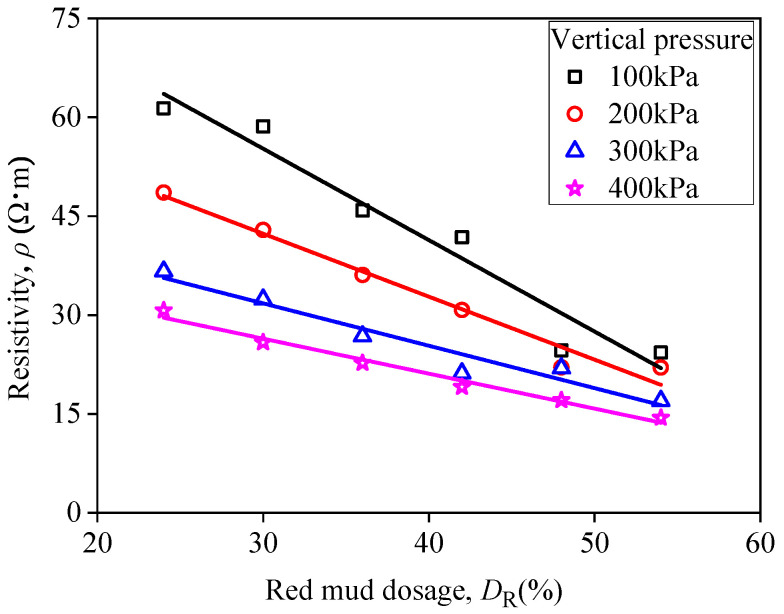
Relationship between resistivity and red mud dosage (under different vertical pressure).

Figure 19 depicts curves of resistivity of RMLS mixture with *D*_R_ under different vertical pressures at the curing age of 3*d*. It is observed that resistivity decreases as *D*_R_ increases and there is a good linear correlation between *D*_R_ and resistivity when a specific vertical pressure is given. In addition, the resistivity decreases as vertical pressure increases when *D*_R_ is fixed. Specifically, the distance between RMLS particles decreases under vertical pressure which will decrease the porosity and increase contact area. In this case, conductive path is shortened and conductive current is increased leading to a decline of complex resistivity amplitude. Furthermore, the amplitude spacing of resistivity decreases as *D*_R_ increases indicating that effect of *D*_R_ on resistivity of RMLS mixture is more significant compared to vertical pressure when *D*_R_ is large.

**Figure 20:**
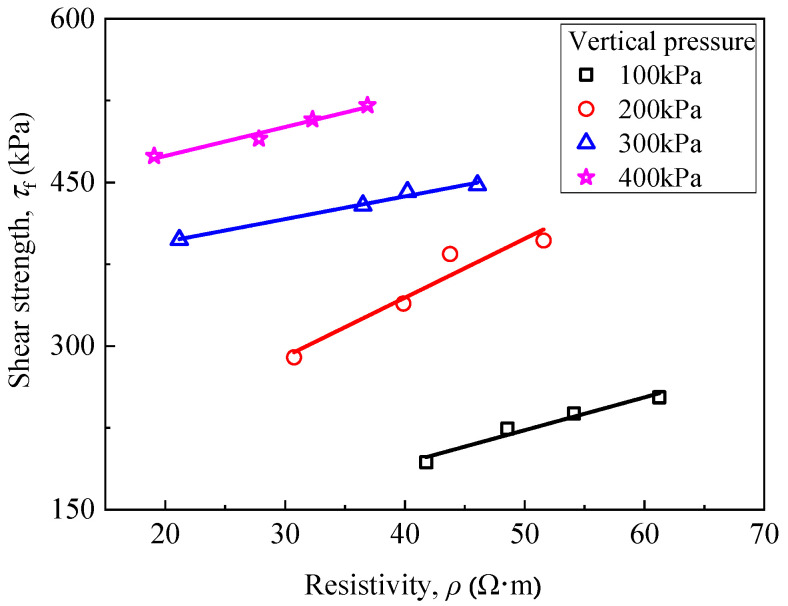
Relationship between resistivity and shear strength (Under different vertical pressure, *D*_R_ = 42%).

A correlation between resistivity and shear strength of RMLS mixture could be predicted owing to the effect of vertical pressure on resistivity as described above. Figure 20 shows the relationship between these two characters, in which shear strength of RMLS mixture increases as the increase of resistivity and a good linear correlation is derived. This result is consistent with previous results of UCS, which again proves that the resistivity can be used as a nondestructive testing technology to characterize the strength performance of soil.

#### 3.5.3. Stress–Strain–Resistivity

**Figure 21:**
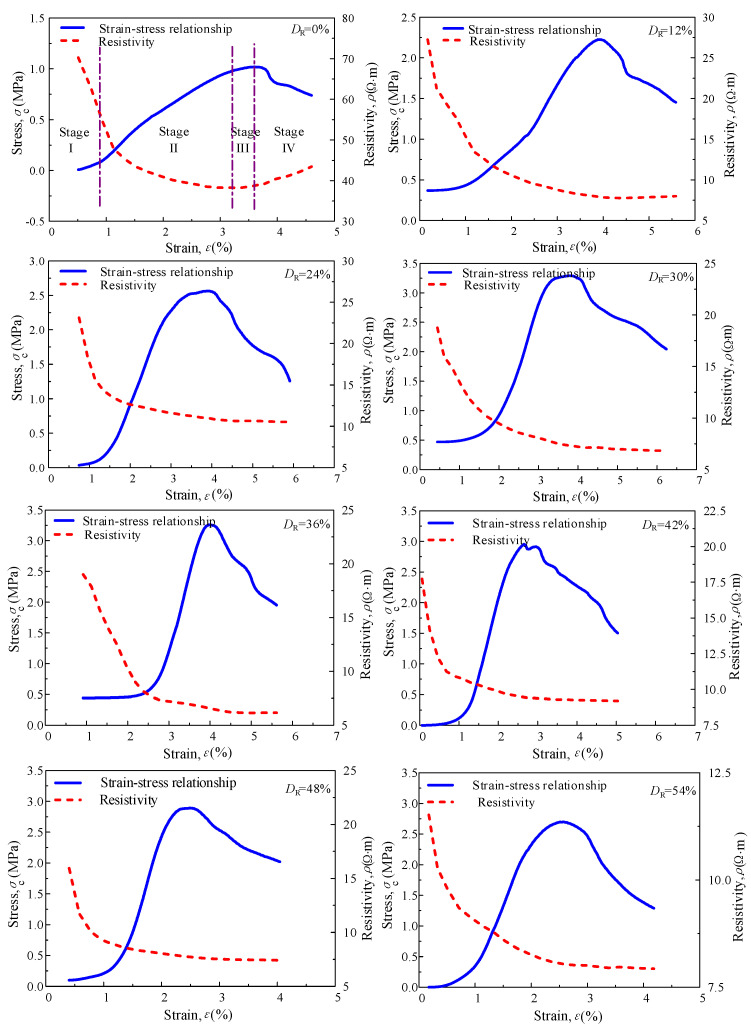
Curves of stress–strain–electrical resistivity with curing age of 14*d*.

To study the stress–strain–resistivity relationship of RMLS mixture during the compression process, change of resistivity was recorded synchronously in the unconfined compression test. A series test result showed that the stress–strain–resistivity curves of RMLS mixture at each curing age exhibit the same pattern and Figure 21 depicts the curve at curing age of 14*d* with different *D*_R_ values. 

The compression process of loessial silt (*D*_R_ = 0) can be divided into three stages (see Figure 21): in stage I (elastic stage), stress of loessial silt increases as the strain increases. Resistivity, however, decreases rapidly showing a negative correlation with stress. When soils particles become more compact, conductive path between solid particle and pore water is shortened and thus resistivity decreases. Although there are subtle cracks in the sample that may affect the resistivity, these cracks are tiny and their influences are negligible. Therefore, resistivity of loessial silt decreases rapidly in stage I. In stage II (strengthening stage), both increasing rate of stress and decreasing rate of resistivity of loessial silt slow down. The minimum value of resistivity is found in this stage. It is apparent that subtle cracks in the sample develop and expand impeding the conductive path, which stops the declining trend of resistivity. In stage III (local deformation stage), stress of loessial silt reaches its maximum value and turns to decrease rapidly when the sample is fractured through and the bearing capacity is lost. While resistivity starts increasing during this period because there is a further expansion of cracks penetrating the sample and blocking the conductivity path. Therefore, the resistivity of loessial silt starts increasing after failure of the sample. 

Note that trends of resistivity of RMLS mixture (*D*_R_ > 0) in elastic and strengthening stages are similar with loessial silt. However, there is no increase of resistivity of RMLS mixture after failure of the sample (in stage III) and the resistivity tends to be a constant value, which is different from loessial silt. Hydration reaction of red mud could explain this result: new cementitious substances generated from red mud connect with soil particles to form a silk and mesh structure. This new stable structure decreases the likelihood of brittle failure for RMLS mixture and the damage form is changed to plastic failure partially. In this case, occurrence of cracks in RMLS sample is reduced and impeding influence on conductivity path is weakened. Therefore, resistivity of loessial silt could remain at a low value after failure of the sample with red mud addition. 

Consequently, it is confirmed that the development and change of cracks in the RMLS mixture under compaction process could be monitored synchronously by nondestructive resistivity test. Analysis in this section may make a contribution to the theory of correlation between resistivity and microstructure.

### 3.6. Microstructure Analysis

#### 3.6.1. SEM Observation

**Figure 22:**
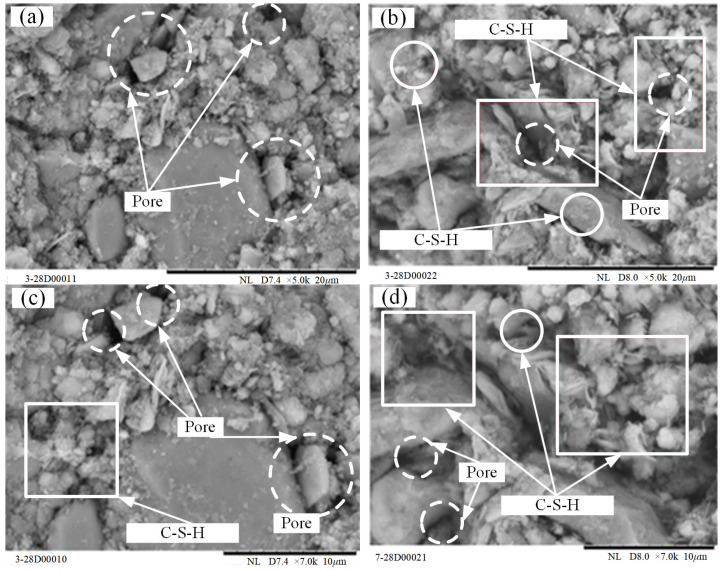
SEM imagines of RMLS after 28*d* curing (**a**) 0% × 5.0 k (**b**) 30% ×5.0k (**c**) 0% × 7.0 k (**d**) 30% × 7.0 k.

Figure 22a,b show the SEM images of loessial silt (*D*_R_ = 0%) and RMLS mixture (*D*_R_ = 30%) respectively after 28 day curing at 5000 times magnification. Figures at 7000 times magnification are also illustrated in Figure 22c,d. A great deal of large pores were observed between the particles of loessial silt leading to a loose surface morphology and weak cementation between large and small particles of loessial silt. Although there are flake and prismatic hydration products between particle pores, the number is too small to fill in these pores. In this condition, the structure of loessial silt is loose and bearing capacity is low.

The microstructure is improved significantly after adding red mud to loessial silt (*D*_R_ = 30%). To be more specific, the number of large particles increases and there is a further development of RMLS mixture crystals. A large number of cluster and needle cementitious materials between large and small particles reduce the number of holes and intensify the cementation between them, which make the soil structure more compact (marked with rectangular box in Figure 22). Flocculated amorphous gels distributed on the surface and surrounding of particles (marked with circular box) can be observed when the magnification of SEM images is increased. However, these flocculent substances are not found in the loessial silt proving red mud addition changes the original hydration reaction and improves the mechanical properties of loessial silt significantly. Therefore, the obviously superior performance of RMLS mixture demonstrates the feasibility of using it as a roadway subgrade material, which is consistent with the analysis of resistivity results.

#### 3.6.2. XRD Analysis

**Figure 23:**
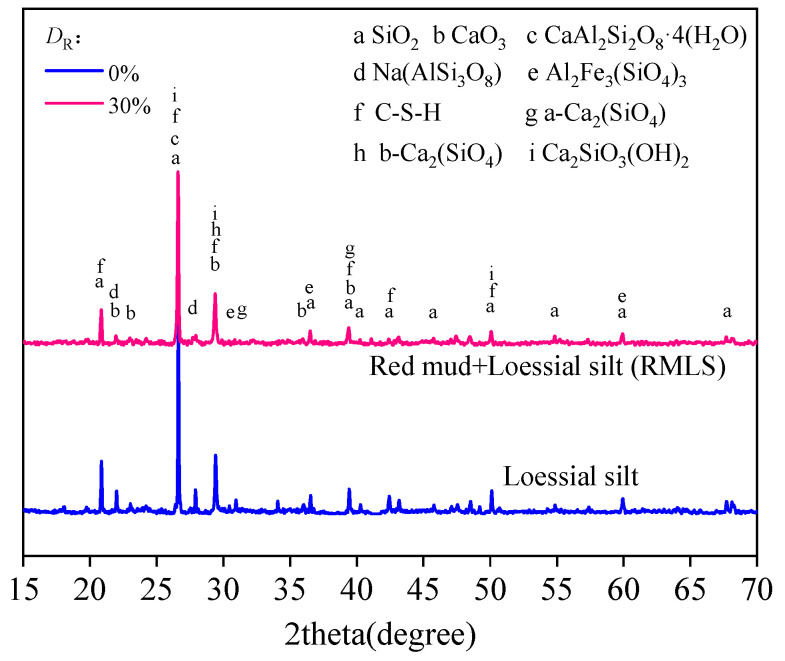
XRD pattern of loessial silt and RMLS for a curing time of 28 days.

Figure 23 shows the XRD patterns of RMLS mixture (*D*_R_ = 30%) and loessial silt. Obvious peaks in loessial silt near 25° and 34° (represent albite and serpentine respectively) disappeared, which is attributed to the fact that alkaline activators and cementitious components in red mud promoted the hydrolysis of active oxides. Specifically, albite and serpentine were decomposed to soluble active ions as Ca^2+^, SiO_4_^4−^, and AlO_2_^−^, which will generate hydration gels [7]. There are some peaks generated from new compounds in RMLS mixture and these hydration products are mainly composed of calcium silicate hydrations (C-S-H), calcium silicate aluminates hydrations (C-A-S-H), and ettringite (Aft) from Figure 23. The peaks corresponding to 2 theta values near 31°, 43.5°, and 48.7° represent C-S-H gel, which is basically consistent with existing study [34]. Aft gel and C-A-S-H gel are observed at 42.5° and 45.5° theta values, respectively [35]. More cementitious materials were formed in RMLS mixture compared to loessial silt because in addition to increased alkalinity of RMLS mixture, red mud can also increase concentration of active ions, which will promote reactions and generations of more gelling hydrates.

## 4. Discussion

**Figure 24:**
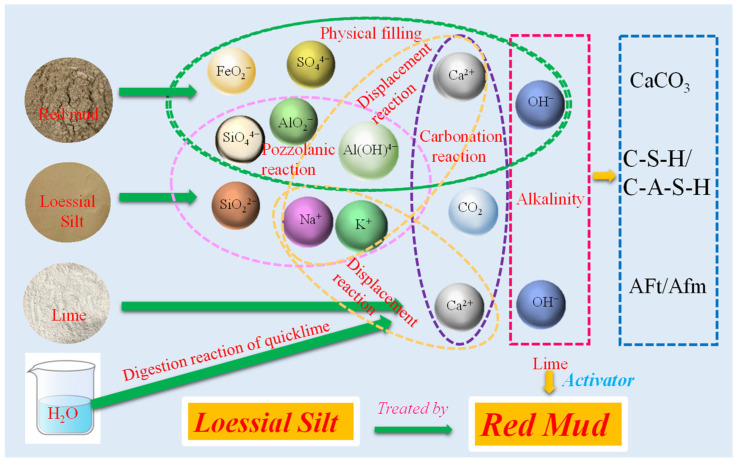
Schematic diagram of the mechanism.

Based on the above microscopic analysis, Figure 24 shows a general schematic diagram of probable interactions in the RMLS mixture. Physical and chemical reactions and formation of sample strength in RMLS mixture are illustrated as follows:

(1) Digestion reaction of quicklime

When quicklime is added into silt, quicklime reacts with water in soil represented by

CaO + H_2_O = Ca(OH)_2_

A large amount of heat is released during this process, which will provide an increasing temperature condition and promote the reactions of Ca(OH)_2_ with other substances in soil to form large amounts of gel products and ettringite, which will provide early strength [36,37].

(2) Pozzolanic reaction

A great deal of OH^−^ ions dissolved from quicklime and red mud creating a strong alkaline environment of pore water, in which, minerals of RMLS mixture, such as albite and nepheline hydrolyzed to SiO_4_^2−^, AlO_2_^−^, and SO_4_^2−^ [38]. Moreover, part of quartz (SiO_2_) reacts slowly in the alkaline environment to form H_3_SiO_4_^−^, H_2_SiO_4_^2−^, HSiO_4_^3−^, and HSiO^−^ [38] while Al_2_O_3_ will also generate Al (OH)_4_^−^, [Al(OH)_6_]^3−^, and AlO_2_^−^ [21,39]. These ions will react with Ca^2+^ decomposed by quicklime in pore water to generate stable products such as C-S-H, C-A-S-H, and Aft. A large amount of OH^−^ is consumed and pH value of the pore water will decline as the reaction progresses until the final equilibrium state is reached. The specific hydration reactions are as follows:

xCa^2+^ + SiO_2_ + nH_2_O → xCaO∙SiO_2_(n + x)∙H_2_O

xCa^2+^ + Al_2_O_3_ + nH_2_O → xCaO∙Al_2_O_3_(n + x)∙H_2_O

xCa^2+^ + y[Al(OH)_6_]^3−^ + zSO_4_^2−^ + nH_2_O → Ca_x_Al_y_(SO_4_)_z_(OH)_6y_∙nH_2_O

(3) Carbonization reaction

CO_2_ dissolves in pore water to form CO_3_^2-^ and then reacts with excess Ca^2+^ to generate CaCO_3_. Due to the good water stability of CaCO_3_, volume of solid particles in the RMLS mixture increase squeezing the surrounding soil particles to make the sample more compact. In this case, strength of RMLS mixture is improved. The specific reaction process is as follows:

CO_2_ + H_2_O → CO_3_^2−^ + H^+^

CO_3_^2−^ + Ca^2+^ → CaCO_3_

(4) Displacement reaction

Ca^2+^ in the pore water of RMLS mixture reacts with Na^+^ and K^+^ generated from active substances to form cementitious substances and improves the mineral composition of the soil skeleton [40]. Meanwhile CaO becomes crystal nucleuses through adhering to the surface of red mud and loessial silt [41]. These crystal nucleuses react with cations in pore water and promote the formation of hydration products.

All of these gels enhance the strength of loessial silt greatly through tightly connecting surrounding particles and hydrates. Moreover, fine red mud particles adhere to the surface of soil particles and fill in the pores between them leading to a more compact and stable structure. However, excess addition of red mud reduces the strength property of RMLS mixture. The OH^−^ dissolved from superfluous red mud leads to early precipitation of gels [42] and thus integrity and stability of soil particle skeleton are reduced. Singh [43] also suggested excess addition of red mud will affect leaching of Al and Si ions, which will impede further hydration reactions and may explain the reduction of strength for RMLS with excess red mud addition. 

Furthermore, there is an agreement with past studies [44,45] regarding using resistivity as a non-destructive testing technique to predict strength performances of soil since change of resistivity value is correlated with the change in microstructure of RMLS mixture. Overall, this study demonstrated the feasibility of using red mud as a stabilization material for roadway subgrade and further investigations need to be performed to explore secondary pollution issue of RMLS mixture for its large scale utilization in subgrade engineering.

## 5. Conclusions

The results are summarized as follows:

(1) The UCS of RMLS mixture first increases and then gradually decreases as the increase of *D*_R_. The *D*_R0_ values corresponding to the maximum value of UCS are found to be 30% to 42% at different curing ages.
(2) Resistivity of RMLS mixture exhibits a decreasing pattern with the increase of *D*_R_. The decreasing process can be divided into three stages in which the descending trend of resistivity is gradually weakened in each stage and tends to be stable finally. A linear relationship was obtained between resistivity and UCS of RMLS mixture. Resistivity can be used as a nondestructive testing technology to evaluate the strength performance of RMLS mixture.
(3) There is a nearly 10 times decrease of coefficient of hydraulic conductivity (*k*) after adding red mud to loessial silt (*D*_R_ = 54%).
(4) Hydration products of RMLS mixture are mainly calcium silicate hydrations (C-S-H), calcium silicate aluminates hydrations (C-A-S-H), and ettringite (Aft).

## Figures and Tables

**Table 1:** Basic physical properties of loessial silt.

Specific Gravity *G*_s_	Maximum Dry Density *ρ*_max_/g·cm^−3^	Optimal Moisture Content *ω*_opt_/%	Liquid Limit *ω*_L_/%	Plastic Limit *ω*_P_/%	Plasticity Index *I*_p_
2.70	1.77	16.7	25.2	15.3	9.9

**Table 2:** Basic physical properties of red mud.

Specific Gravity *G_s_*	Liquid Limit *ω_L_*/%	Plastic Limit *ω_P_*/%	Plasticity Index *I_p_*
2.71	47.3	35.6	11.7

**Table 3:** Main chemical composition of red mud.

**Composition**	SiO_2_	Al_2_O_3_	CaO	Na_2_O	Fe_2_O_3_	Others
**Percentage/%**	26.80	24.03	15.58	7.15	6.96	19.48

**Table 4:** Heavy metals leaching toxicity in red mud.

Element	RM/(mg kg^−1^)	Concentration Limit/(mg L^−1^)	Leaching Concentration/(mg L^−1^)
Pb	86	5	0.0018
Cr	240	15	0.0134
Cd	6	1	0.000166
Ni	64	5	0.000326
Be	4.97	0.02	0.000084
As	8.88	5	0.023
F^−^	1275	100	100

**Table 5:** Test plans and the compaction test results.

Loessial Silt/%	Lime/%	Red Mud/%	Optimal Moisture Content/%	Maximum Dry Density/g/cm^3^
100	10	0	22.0	1.96
100	10	6	23.0	1.95
100	10	12	23.8	1.94
100	10	18	24.8	1.93
100	10	24	25.6	1.92
100	10	30	26.4	1.91
100	10	36	27.2	1.90
100	10	42	28.1	1.89
100	10	48	29.0	1.88
100	10	54	29.9	1.87

**Table 6:** Parameters of unconfined compressive strength with the curing age.

Red Mud Dosage (*D*_R_)/%	*a*	*b*	*R* ^2^
6	0.6262	0.0309	0.9661
12	0.5877	0.6655	0.9991
18	0.7769	0.5167	0.8495
24	0.827	0.6035	0.9006
30	1.2263	0.0274	0.994
36	0.9958	0.5667	0.9818
42	0.5169	1.7752	0.8451
48	0.3025	2.1436	0.9386
54	0.4082	1.4293	0.9513

**Table 7:** Parameter values for the fitting UCS equation.

*q*_u0_	*B*	*C*	*D*
3.284	−3.795	17.191	26.627

**Table 8:** Values of *A*, *B*, *C* for hydraulic conductivity coefficient expression.

Osmotic Pressures, *P*	*A*	*B*	*C*	Coefficient of Determination, *R*^2^
50 kPa	16.326	−2.205	−3.000	0.997
100 kPa	36.018	−5.282	−2.967	0.993
150 kPa	44.848	−8.027	−2.826	0.994
200 kPa	48.399	−8.250	−2.563	0.999
250 kPa	51.038	−9.251	−1.465	0.996

## Data Availability

The data used to support the findings of this study are available from the corresponding author upon request.
